# First-Principles Calculations of the Mechanical Properties of Doped Cu_3_P Alloys

**DOI:** 10.3390/ma17071677

**Published:** 2024-04-05

**Authors:** Xiao Ma, Fang Cheng, Weiqing Huang, Lian He, Zixin Ye, Shimeng Yu, Ling Hu, Dingkun Yu, Hangyan Shen

**Affiliations:** 1 College of Materials and Chemistry, China Jiliang University, Hangzhou 310018, China; 2 Hangzhou Huaguang Advanced Welding Materials Co., Ltd., Hangzhou 311112, China; 3 Zhejiang Zhekan Testing Co., Ltd., Ningbo 315033, China; 4 Liangxin Honors College, China Jiliang University, Hangzhou 310018, China

**Keywords:** mechanical properties, first-principles calculation, density of states

## Abstract

In the quest to enhance the mechanical properties of CuP alloys, particularly focusing on the Cu_3_P phase, this study introduces a comprehensive investigation into the effects of various alloying elements on the alloy’s performance. In this paper, the first principle of density universal function theory and the projection-enhanced wave method under VASP 5.4.4 software are used to recalculate the lattice constants, evaluate the lattice stability, and explore the mechanical properties of selected doped elements such as In, Si, V, Al, Bi, Nb, Sc, Ta, Ti, Y and Zr, including shear, stiffness, compression, and plasticity. The investigation reveals that strategic doping with In and Si significantly enhances shear resistance and stiffness, while V addition notably augments compressive resistance. Furthermore, incorporating Al, Bi, Nb, Sc, Ta, Ti, V, Y, and Zr has substantially improved plasticity, indicating a broad spectrum of mechanical enhancement through precise alloying. Crucially, the validation of our computational models is demonstrated through hardness experiments on Si and Sn-doped specimens, corroborating the theoretical predictions. Additionally, a meticulous analysis of the states’ density further confirms our computational approach’s accuracy and reliability. This study highlights the potential of targeted alloying to tailor the mechanical properties of Cu_3_P alloys and establishes a robust theoretical framework for predicting the effects of doping in metallic alloys. The findings presented herein offer valuable insights and a novel perspective on material design and optimization, marking a significant stride toward developing advanced materials with customized mechanical properties.

## 1. Introduction

**Figure 1:**
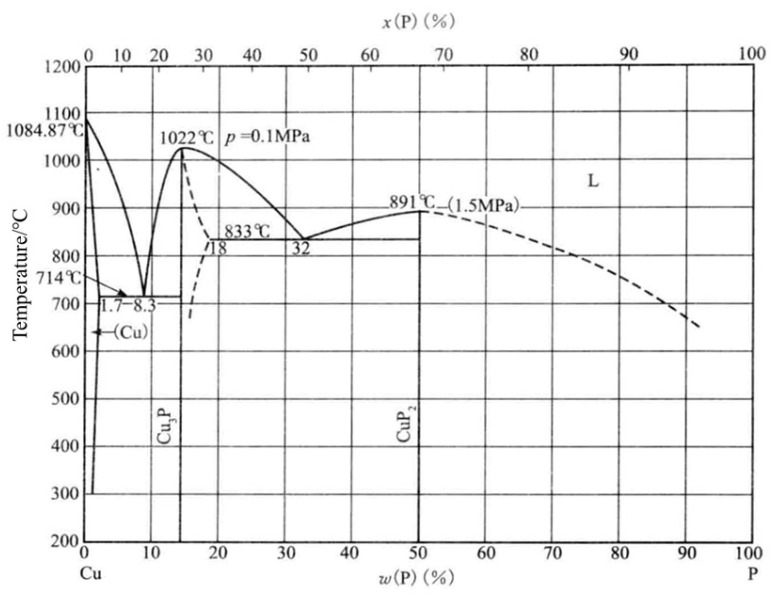
Binary phase diagram of Cu-P alloy [3].

Copper–phosphorus brazing alloys are a class of brazing filler metals mainly composed of copper (Cu) and phosphorus (P), which are widely used in aerospace, electronics, energy, transportation, military, automotive, and other industries due to their excellent performance [1,2]. The binary phase diagram of Cu-P alloys is shown in Figure 1 [3], showing that Cu_3_P is the main copper phosphide compound and one of the main phosphorus compounds in copper-based brazing materials. Using a combination of thin-film experiments, electronic structure calculations, and semiclassical Boltzmann transport theory, Crovetto et al. found that, ideally, Cu_3_P is a semiconductor with a small overlap between valence and conduction bands. Experimentally realizable Cu_3_P films always behave as p-type semimetals with natural doping due to copper vacancies [4]. However, a large amount of Cu_3_P compounds leads to poor plasticity at room temperature [5]. Adding various alloying elements is one of the most common and effective methods researchers use to improve the brazing performance of Cu-P brazing materials [6,7]. The effect of Ag addition on Cu-P brazing materials was investigated by Tan-Dong Xia et al. [8]. Elemental silver addition was carried out on five different types of Cu-P brazing materials, and the results showed that the brittleness problem of the joints improved with the increase in silver content. The modification of copper–phosphorus brazing materials was intensively investigated by Huang Junlan et al. [9]. The results of his work showed that the addition of elemental Sn to copper–phosphorus brazing materials also significantly lowered their melting points while improving their wetting properties, but still did not solve the brittleness problem, which is a common concern in the commercial world. Li Shuzhen [10] added three elements, Fe, Mn, and Zn, to copper–phosphorus brazing materials and thoroughly explored their effects on the comprehensive performance of copper–phosphorus brazing materials. The results show that the addition of Fe should not exceed 0.1 wt.%, and when the addition is more significant than this value, the tensile strength of brazed joints decreases, and porosity appears in the tensile fracture. The addition of more than 1.5 wt.% of Mn elements will produce a MnP and Mn_2_P phase (black worm-like organization) and will also reduce the tensile strength of brazed joints; when Zn content is more than 2.0 wt.%, the joint tensile strength is reduced, the tensile fracture weave becomes shallow, and the mechanical properties significantly deteriorate.

The influence of various microalloying elements on the mechanical properties of copper–phosphorus brazing materials is still the research focus in this field. However, due to the long cycle time and low efficiency of traditional experiments, computational materials science has become an emerging discipline covering computer and materials science [11,12], which simplifies the research process by calculating the doping of Cu-P brazing materials through the first nature principle, which can screen out the elements that satisfy the properties for experimental verification [13]. In this paper, we choose to add the elements of group 3A (Al and In), group 4A (Si, Ge, Sn, and Pb), group 5A (Sb and Bi), group 3B (Sc and Y), group 4B (Ti, Zr, and Hf), and group 5B (V, Nb, and Ta) into the Cu_3_P phase, in which many compounds of As contain toxicity, and therefore are excluded in this paper. In this paper, the effect of doping before and after the first principle calculations is studied. The second part of this paper calculates the formation energies before and after doping and identifies the atoms replaced by doping. The third part shows the calculations of the mechanical properties and proves the correctness of the calculations by hardness experiments of doped Si and Sn. It also explains the mechanical properties by the density of states, and the fourth part concludes the paper.

## 2. Crystal Structure and Calculation Methods

### 2.1. Crystal Structure

**Figure 2:**
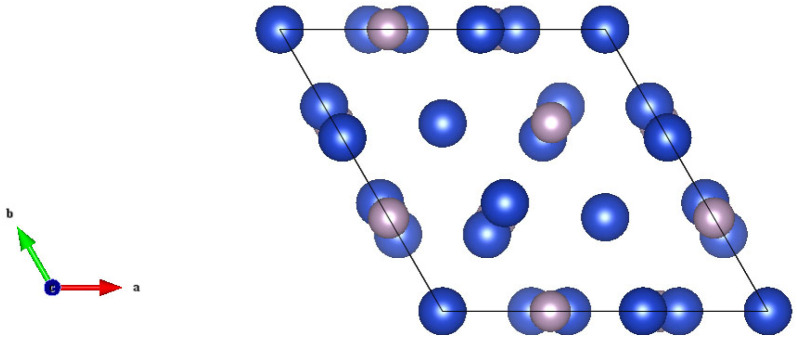
The crystal structure of Cu_3_P (*P*6_3_*cm*, No.185).

It is necessary to establish the structural model according to the experimental or database parameters. Cu_3_P belongs to the hexagonal crystal system; there are 24 atoms in the calculation model, which contains 18 Cu atoms and 6 P atoms, and the established crystal structure model is shown in Figure 2. The blue one is a Cu atom and the other one is a P atom.

The doping is modeled by replacing atoms, and for the convenience of calculation, only one site of Cu or P atom is replaced in the bulk phase Cu_3_P, and the formation energy after doping other atoms can be calculated by Equation (1) [14]. The calculation results are shown in Table 1.

(1) $$E=E_{total}-E_{Cu_{3}P}+aE_{x}-aE_{y}$$

where $E_{total}$ is the total energy of the system after doping, $E_{Cu_{3}P}$ is the total energy of Cu_3_P before doping, $E_{x}$ and $E_{y}$ are the energies of the doped and replaced free atoms, respectively, and a is the number of atoms to be replaced. All calculations in this paper are performed using the Projected-Augmented Wave (PAW) method under the VASP software based on density generalized function theory (DFT), and Encut-off is set to 500 eV and K points are set to 6 × 6 × 6. Section 2.2, Calculation Methods and Parameters, is for details. And for ease of viewing, all doping chemical formulae in this paper are expressed using three elements, e.g., the chemical formula of CuPAl actually represents Cu_18_P_5_Al or Cu_17_P_6_Al.

**Figure 3:**
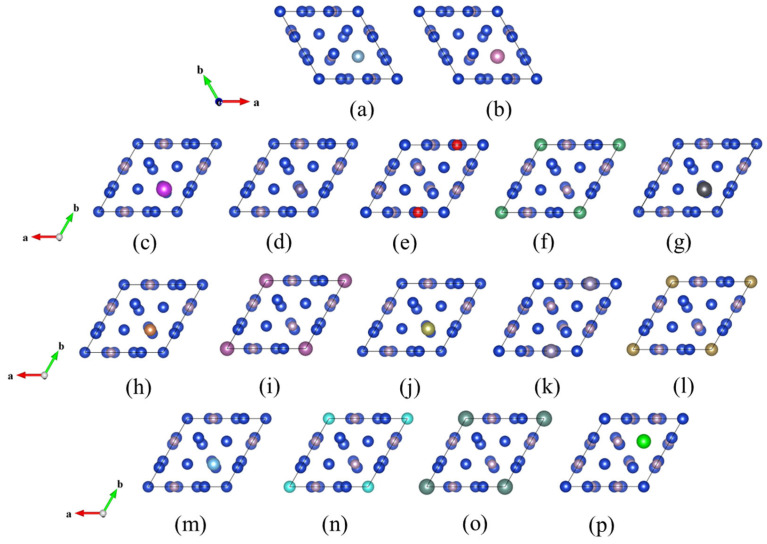
Crystal structure after doping of (**a**) CuPAl, (**b**) CuPIn, (**c**) CuPBi, (**d**) CuPGe, (**e**) CuPHf, (**f**) CuPNb, (**g**) CuPPb, (**h**) CuPSb, (**i**) CuPSc, (**j**) CuPSi, (**k**) CuPSn, (**l**) CuPTa, (**m**) CuPTi, (**n**) CuPV, (**o**) CuPY, and (**p**) CuPZr.

From Table 1, the doping formation energy of Bi, Ge, Hf, Pb, Sb, Si, Sn, and Ti atoms replacing P atoms is lower than the doping formation energy of replacing Cu atoms, while the doping formation energy of Al, In, Nb, Sc, Ta, V, Y and Zr atoms replacing Cu atoms is lower than the doping formation energy of replacing P atoms, and the smaller the doping formation energy, the more stable the structure is, so the crystal structure after replacing one P atom with Bi, Ge, Hf, Pb, Sb, Si, Sn and Ti atoms and one Cu atom with Al, In, Nb, Sc, Ta, V, Y and Zr atoms is shown in Figure 3. The colors of the Cu and P atoms are consistent with those in the Cu3P model, leaving the other one as a doped atom.

### 2.2. Calculation Methods and Parameters

When studying the interaction between electrons and nuclei, the calculation of the PAW pseudopotential is usually more accurate than the super-soft pseudopotential [15]. The PAW pseudopotential is used in quantum mechanical calculations to treat electron-nucleus interactions. This method is commonly used to calculate the electronic structure of materials, such as density-functional theory, to more efficiently describe the behavior of electrons in the vicinity of atomic nuclei. Therefore, all calculations in this paper are performed using the Projected-Augmented Wave (PAW) method under the VASP software based on density generalized function theory (DFT). For Cu_3_P, among the three pseudopotentials, PAW-PBE, PAW-LDA, and PAW-PBEsol, the PAW-PBE calculation is the most reliable.

**Figure 4:**
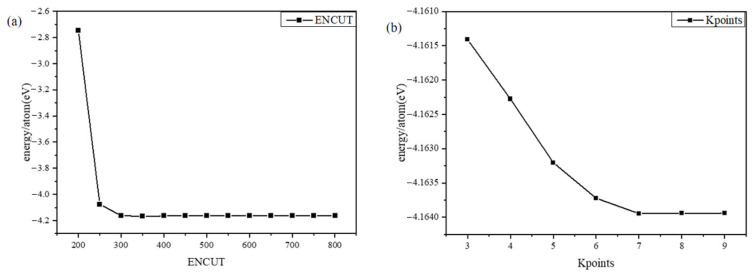
Convergence test results: (**a**) ENCUT test results, (**b**) K points test results.

In order to balance the speed and accuracy of the calculation, two parameters, Ecut-off and K points, are tested [16]. The purpose of the convergence test is to reduce the error due to the artificial setting of the parameters by calculation. The results of the truncation energy Ecut-off, as well as the K points test, are shown in Figure 4.

The system energy reaches convergence when the individual atomic energy difference of the system is less than one meV. According to Figure 4, it can be seen that the convergence condition can be satisfied when Encut-off is taken as 500 eV and K points 6 × 6 × 6 are taken, and the convergence criterion of 10^−5^ eV is used in the calculation, and all the force values of the structure are required to reach the convergence threshold of 0.001 eV∙A^−1^.

## 3. Results

### 3.1. Lattice Constants

**Figure 5:**
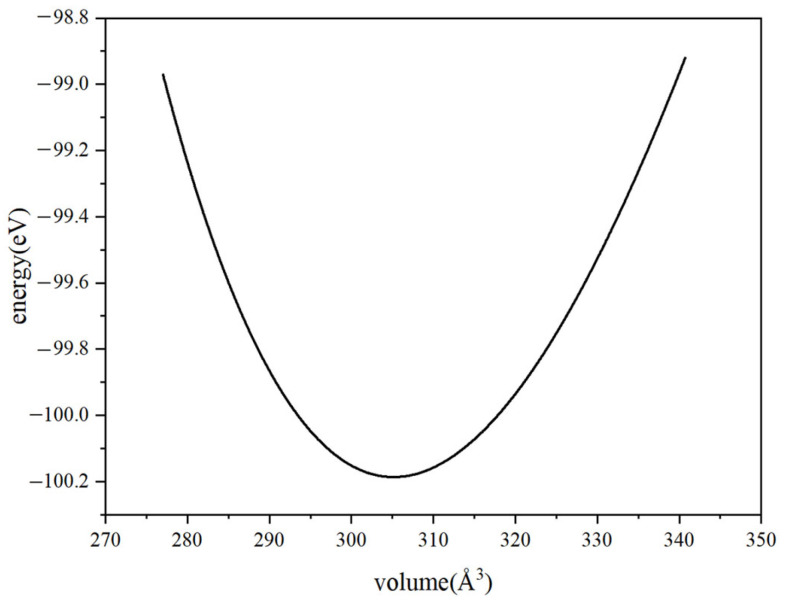
The relationship between the total energy of primitive cell and volume.

After establishing the crystal structure model, the geometric structure of Cu_3_P was first optimized to obtain its stable structure. Figure 5 shows the variation of the total energy of the primary cell with volume, i.e., the E-V curve, and the value corresponding to the lowest energy point is the calculated value of the lattice constant. The values of the lattice parameters obtained after calculation are shown in Table 2, and we compared them with the experimental values and other literature-calculated lattice parameter values. We observe that our calculations agree with previous ones.

The geometry of the doped structure shown in Figure 3 was optimized, and the comparison of the lattice constant results after doping with Al, In, Si, Ge, Sn, Pb, Sb, Bi, Sc, Y, Ti, Zr, Hf, V, Nb and Ta atoms are shown in Table 3.

As can be seen from Table 3, the lattice constants and volumes after doping slightly increase compared with those before doping. This is due to the lattice distortion of the atomic structure caused by doping these elements. The atomic radii of Cu, P, Al, In, Bi, Ge, Hf, Nb, Pb, Sb, Sc, Si, Sn, Ta, Ti, V, Y, and Zr are 140 pm, 98 pm, 160 pm, 167 pm, 155 pm, 122 pm, 159 pm, 146 pm, 175 pm, 140 pm, 160 pm, 111 pm, 145 pm, 146 pm, 147 pm, 153 pm, 212 pm, and 206 pm, respectively. According to the quantum chemical theory, the radii of the atoms of Bi, Ge, Hf, Pb, Sb, Si, Sn, and Ti are larger than that of the atom of P. The radii of Al, In, Nb, Sc, Ta, V, Y, and Zr atoms are larger than those of Cu atoms, so the cell will expand after the dopant atoms enter the lattice. The interatomic distance will increase, resulting in a corresponding increase in volume.

For any spontaneous process, the change in Gibbs free energy (ΔG) must be negative (ΔG < 0) at constant temperature and pressure. If ΔG = 0, the system is in equilibrium; if ΔG > 0, the process is non-spontaneous under the given conditions, indicating that the direction of the reaction is thermodynamically unstable. Thus, by calculating the ΔG of a chemical reaction or physical process, it is possible to determine whether the reaction tends to occur or not, which directly correlates to the thermodynamic stability of the substance.

(2) G=H−TS

where *G* is Gibbs free energy, *H* is enthalpy, *T* is thermodynamic temperature, and *S* is entropy.

**Figure 6:**
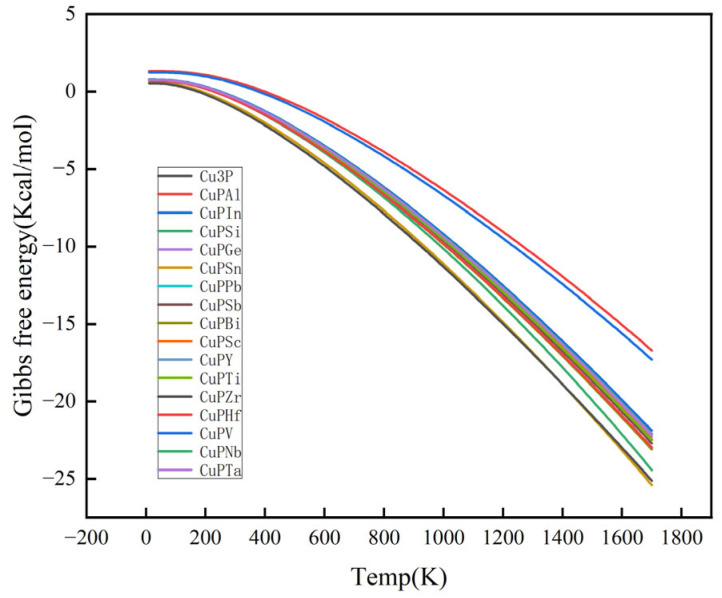
Comparison of b Gibbs free energy before and after element doping.

As can be seen in Figure 6, at high temperatures, the Gibbs free energies of all doped systems are greater than 0, indicating that all systems are thermodynamically stable.

### 3.2. Mechanical Properties

According to the Voigt–Reuss–Hill theory [19], it is known that the upper limits ($B_{V}$, $G_{V}$) can be obtained by the Voigt model, the lower limits ($B_{R}$, $G_{R}$) by the Reuss model, and the $B_{H}$, $G_{H}$ obtained by the Hill model are average values, which are more in line with the actual performance. The calculation formula is as follows:

(3) $$B_{V}=2(C_{11}+C_{12}+C_{33}/2+2C_{13})/9$$

(4) $$B_{R}={\;[(C}_{11}+C_{12})C_{33}-2C_{13}^{2}]/(C_{11}+C_{12}+{2C}_{33}-4C_{13})$$

(5) $$G_{V}=(7C_{11}-5C_{12}+2C_{33}-4C_{13}+12C_{44})/30$$

(6) $$G_{R}=5[{(C}_{11}+C_{12})C_{33}-2C_{13}^{2}](C_{11}-C_{12})\;C_{44}/2\left\{3B_{V}C_{44}\left(C_{11}-C_{12}\right)+[{(C}_{11}+C_{12})C_{33}-\;2C_{13}^{2}](C_{11}-C_{12}+2\;C_{44})\right\}$$

The Poisson’s ratio (v) is calculated as follows:

(7) $$v=(3B_{H}-2G_{H})/2(3B_{H}+G_{H})$$

**Figure 7:**
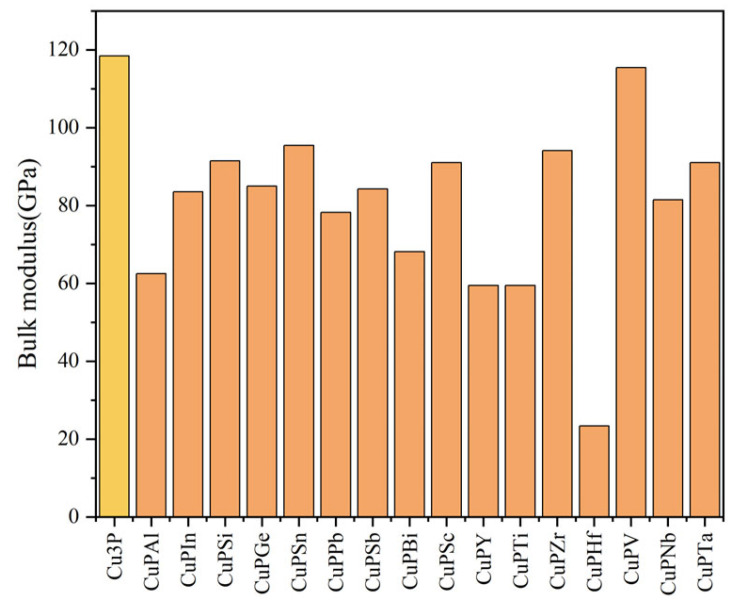
Comparison of bulk modulus before and after doping elements.

**Figure 8:**
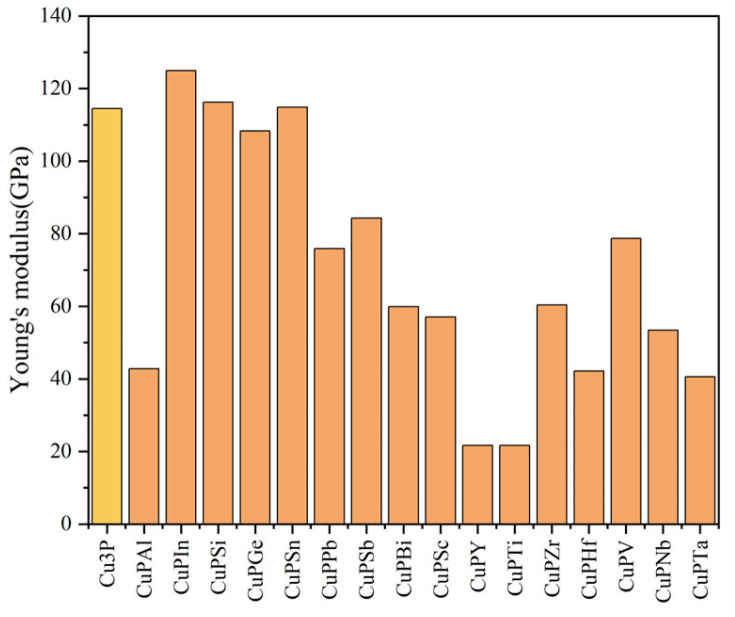
Comparison of Young’s modulus before and after doping elements.

**Figure 9:**
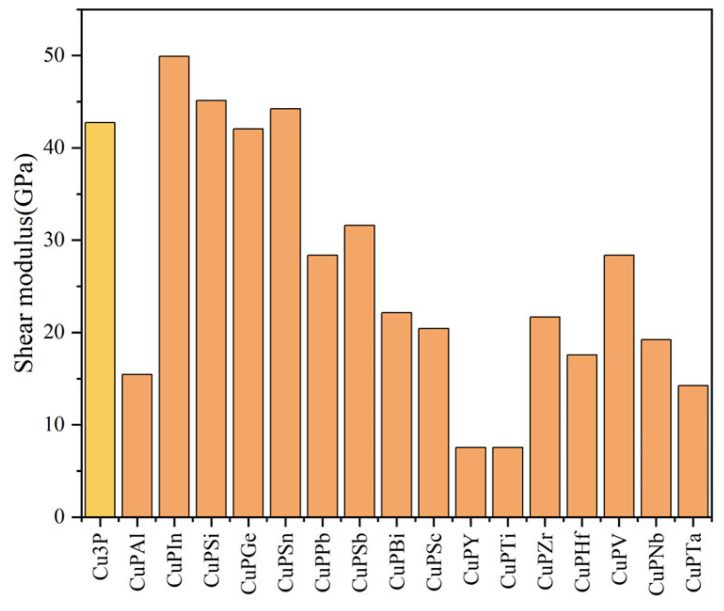
Comparison of shear modulus before and after doping elements.

**Figure 10:**
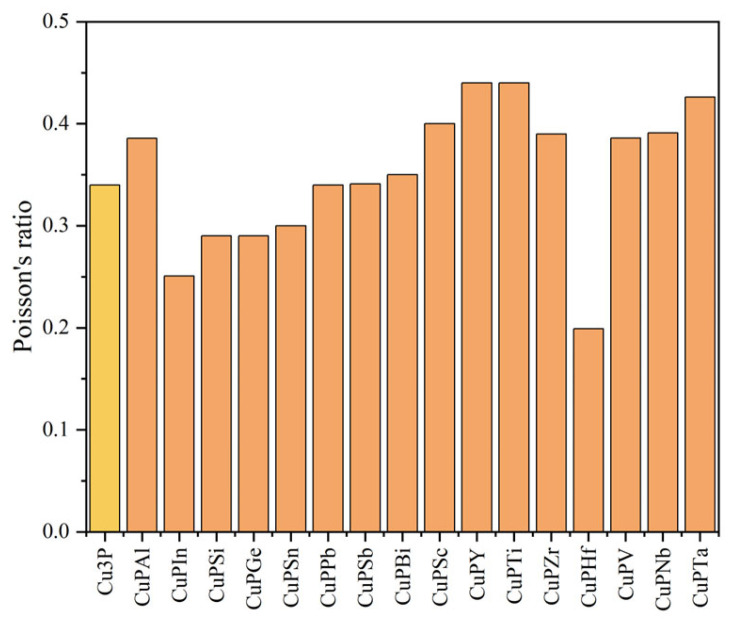
Comparison of Poisson’s ratio before and after doping elements.

The values obtained from the calculations based on Equations (3)–(7) are shown in Figure 7, Figure 8, Figure 9 and Figure 10. The bulk modulus is the ratio of the compressive stress applied to the material. The material is subjected to body stress and is compressed to produce body strain, and the ratio of body strain to body stress is adjusted [20]. The larger the bulk modulus, the stronger the compression resistance, and the order of compression resistance before and after doping is Cu_3_P > CuPV > CuPSn > CuPZr > CuPSi > CuPSc > CuPTa > CuPGe > CuPSb > CuPIn > CuPNb > CuPPb > CuPBi > CuPAl > CuPTi > CuPY > CuPHf. Young’s modulus is the ratio of stress to strain characterizing the uniaxial stress to the material, and the larger the Young’s modulus, the greater the stiffness of the material, and the order of stiffness before and after doping is CuPIn > CuPSi > CuPSn > Cu_3_P > CuPGe > CuPSb > CuPV > CuPPb > CuPZr > CuPBi > CuPSc > CuPNb > CuPAl > CuPHf > CuPTa > CuPTi > CuPY. The shear modulus is the ratio of the tangential strain to the tangential stress produced by applying shear stress to the material within the elastic strain [20]. The greater the shear modulus, the stronger the shear resistance. The order of shear resistance is CuPIn > CuPSi > CuPSn > Cu_3_P > CuPGe > CuPSb > CuPPb > CuPV > CuPBi > CuPZr > CuPSc > CuPNb > CuPHf > CuPAl > CuPTa > CuPTi > CuPY. Poisson’s ratio (*v*) can reflect the elastic properties and plasticity of the material. In general, for values in the range of −1 to 0.5, the greater its value, the better the elasticity and material plasticity [21]. Enhancing plasticity and elasticity in brazing materials improves the filler and adaptability of the weld, reduces the effects of thermal stresses, and improves the quality of the weld. It can be seen from Figure 10 that doping Ti, Y, Ta, Sc, Nb, Zr, V, Al, Bi, and Sb can improve the plasticity and elasticity; the doping of Pb, Sn, Si, Ge, In, and Hf will make Cu_3_P brittle.

### 3.3. Hardness

Hardness is one of the most basic tests to examine mechanical properties and refers to the ability of a material to resist invasion of the surface by external forces. Hardness testing can explain how Cu_3_P will likely behave when used as an engineering material. For example, knowing the hardness of a material is critical in the manufacture of precision parts or in industrial applications that require highly wear-resistant materials. The material hardness is closely related to the bulk and shear modulus [22], and the material hardness is determined by the following equation [23,24]:

(8) $$Hv=2{\left(K^{2}G_{H}\right)}^{0.585}-3$$

where $K=G_{H}/B_{H}$.

**Figure 11:**
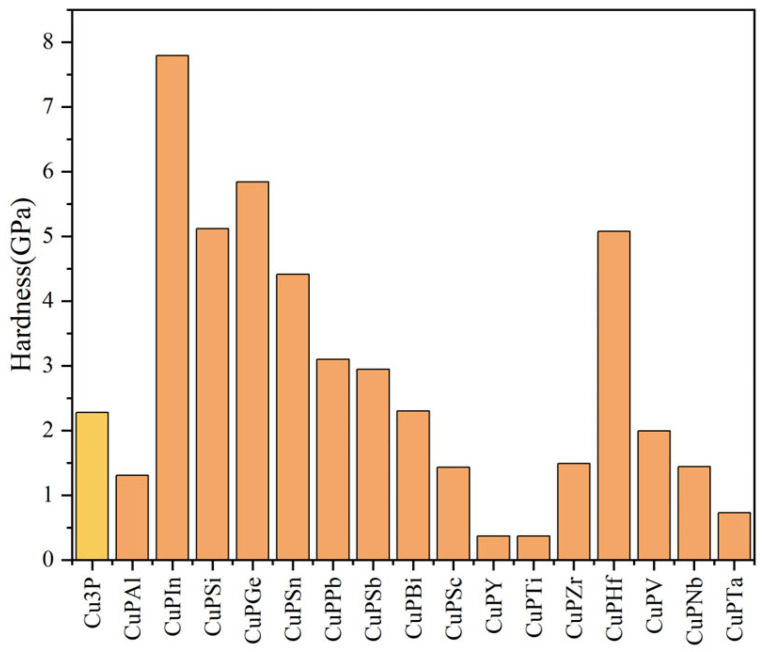
Hardness calculation value before and after doping elements.

The magnitude of hardness is the physical quantity that best proves the strength of the mechanical properties. As can be seen from Figure 11, the hardness increases after doping with In, Bi, Ge, Hf, Pb, Sb, Si, and Sn atoms, and the hardness experiments of doped Si and Sn are in full agreement with the experimental test results, while the hardness decreases after doping with Al, Nb, Sc, Ta, Ti, V, Y, and Zr atoms. The hardness and plasticity of metals are interlinked, and the greater the hardness, the weaker the plasticity, which is consistent with the conclusions drawn from Poisson’s ratio in Section 3.3.

In this paper, we selected two elements, Si and Sn, to prepare samples for experimental comparison based on the mass ratio after doping. A comparison of the actually measured composition and the calculated modeled composition content is shown in Table 4, where those outside the parentheses are the actual ICP-measured contents, and those inside the parentheses are the calculated modeled constituent contents.

**Figure 12:**
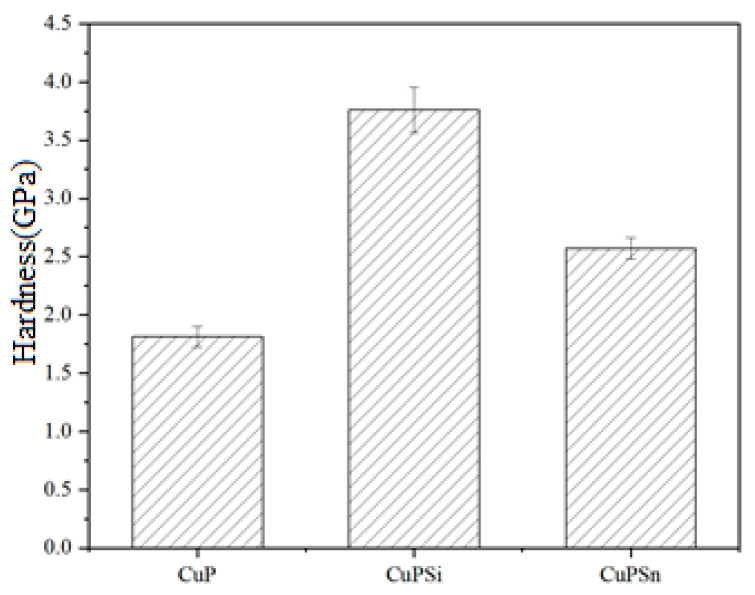
Hardness experimental value before and after doping elements.

The hardness experiment is as follows: firstly, cut the melted brazing material into small pieces and use the automatic setting machine ZXQ-1(Shanghai Jinshang Machinery Equipment Co., Shanghai, China) to set it. After that, use 400#, 800#, 1500#, and 2000# sandpaper to polish on the metallographic pre-grinding machine 1M-1 (Shanghai Weiwei Electronic Technology Co., Shanghai, China) in turn; then, use the metallographic polishing machine PG-2A (Shanghai Jinshang Machinery Equipment Co., Shanghai, China), and use the polishing paste to polish the surface into a smooth mirror, and lastly, press the microhardness tester HVS-1000 (Shanghai Kaite Instrument Co., Shanghai, China) indenter into the surface of the object. During the testing process, the test force was selected as HV0.2, the holding time was set to 30 s, 8–10 points were tested for each sample, and the average value of the microhardness test for each sample was the final result. It can be seen from Figure 12 that the hardness increases after doping with Si and Sn atoms, which is in full agreement with the hardness calculation results. The hardness and plasticity of the metal are interconnected; the greater the hardness, the weaker the plasticity, which is consistent with the conclusion drawn from Poisson’s ratio in Section 3.2.

### 3.4. PDOS and TDOS

**Figure 13:**
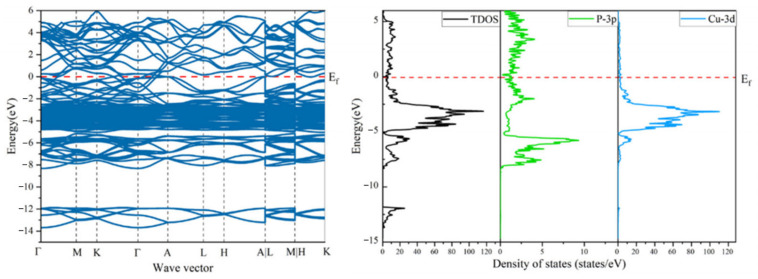
Calculated band structure and density of states of bulk Cu_3_P.

Firstly, the electronic band structure and state density for Cu_3_P in its bulk form were computed, as illustrated in Figure 13. This computational outcome is in alignment with prior findings. Typically, electronic structures are representable through two distinct perspectives: the density of states (DOS) and the band structure. Contributions to the DOS arise from the Cu-3d and P-3s electronic states. For the bulk Cu_3_P, electrons within the P atom’s 3s orbital predominantly fill the energy range of −14 eV to −12 eV. Meanwhile, electrons in the Cu atom’s 3d orbital primarily fill the −5 eV to 0 eV energy spectrum. The band spectrum appears quite compact, suggesting a localized nature for Cu’s 3d orbitals, a trait typically observed in transition metals.

PDOS (Partial Density of States) and TDOS (Total Density of States) are key concepts used in solid-state physics and computational chemistry to characterize the electronic structure of a material. PDOS represents the density of electronic states in specific atomic orbitals in a solid and helps to understand the contribution of different orbitals to the electronic structure. In contrast, TDOS describes the total density distribution of all electronic states in a given energy range, providing information about the overall electronic structure of a material.

**Figure 14:**
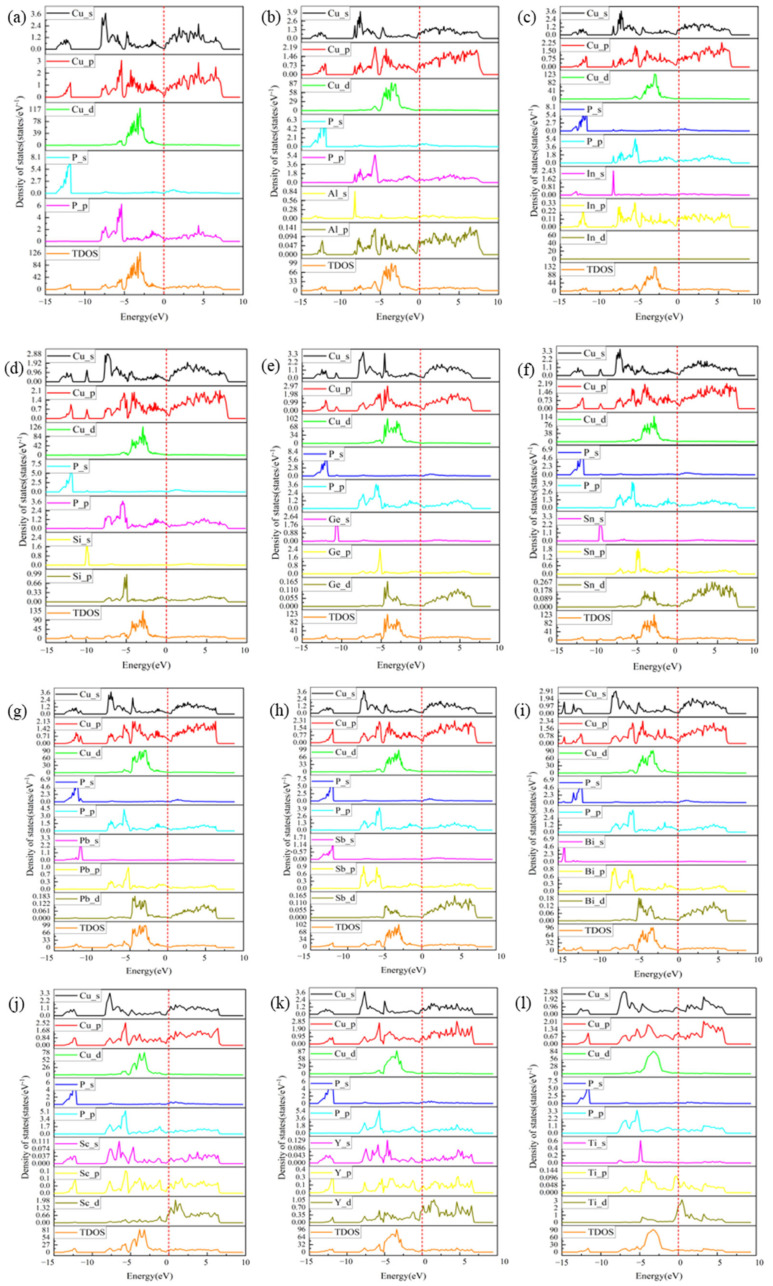

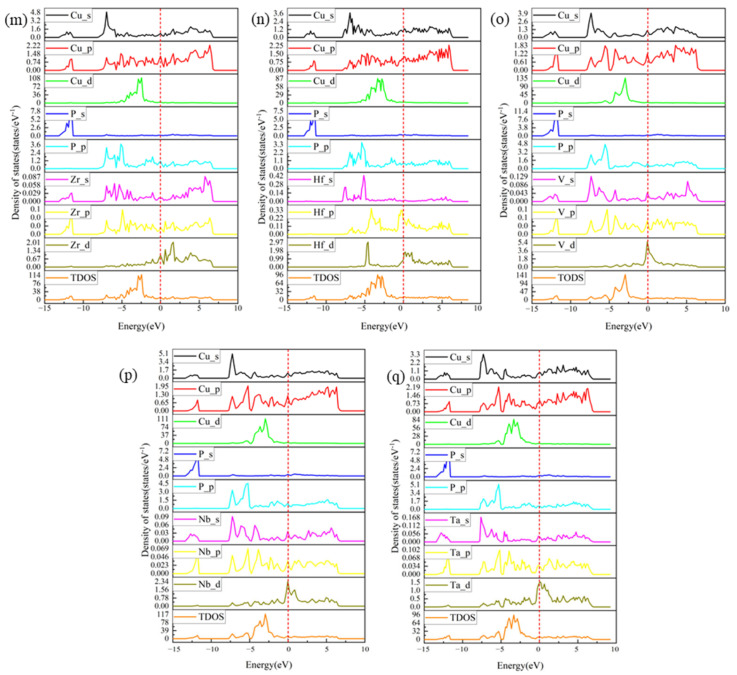
PDOS and TDOS of (**a**) CuP, (**b**) CuPAl, (**c**) CuPIn, (**d**) CuPSi, (**e**) CuPGe, (**f**) CuPSn, (**g**) CuPPb, (**h**) CuPSb, (**i**) CuPBi, (**j**) CuPSc, (**k**) CuPY, (**l**) CuPTi, (**m**) CuPZr, (**n**) CuPHf, (**o**) CuPV, (**p**) CuPNb, and (**q**) CuPTa.

The DOS in the range of −8 eV~−2 eV is mainly contributed by the p- and d-orbitals, and thus p-d orbital hybridization occurs. The more coupling between the p-electronic direction of the nonmetal and the d-electronic direction of the metal, the more covalent bonds are formed, which resists the shear strain, and the stiffer the system is, as can be seen from Figure 14c–f. It can be seen that the coupling of doped In, Si, Sn, and Ge in the range of −8 eV~−2 eV is the highest, and the energy in this range after doping is significantly higher than that before doping, which is consistent with the shear modulus and hardness calculated in this paper. DOS in the range of 2 eV to 8 eV is mainly contributed by the d orbitals, which mainly form metallic bonds. From Figure 14j–m,p,q, it can be seen that the energy fluctuation of the doped TDOS is larger than that of the undoped TDOS, which indicates that the d-orbitals of Sc, Y, Ti, Zr, Nb, and Ta are contributing more to the system, which can easily form a metallic bond with CuP and reduce the hardness of the system. This is in line with the trend calculated in Section 3.3.

## 4. Conclusions

In this paper, we doped Al, In, Si, Ge, Sn, Pb, Sb, Bi, Sc, Y, Ti, Zr, Hf, V, Nb, and Ta atoms into the Cu_3_P crystal structure by the doping method of replacing atoms, and obtained the lattice constants, lattice stability, and mechanical properties of Cu_3_P as well as after doping based on the first-principles calculation method of DFT using PAW pseudopotential under VASP software. The conclusions are as follows:

(1) The structural stability is in the order of CuPSi > CuPZr > CuPTa > CuPNb > CuPV > CuPSc > CuPGe > CuPTi > CuPHf > CuPY > CuPSb > CuPPb > CuPAl > CuPBi > CuPSn > CuPIn, and the Si-doped structure is the most stable, and the calculated binding energy (*E_coh*) and enthalpy of generation (Δ*H*) are negative, indicating that the formation process of the compound is exothermic and can be formed spontaneously. The Gibbs free energy of all systems is less than 0, indicating that all doped compounds are thermodynamically stable.

(2) The doping of In and Si can effectively improve the shear resistance and stiffness of Cu_3_P, the doping of V can improve the compression resistance, and the doping of Al, Bi, Nb, Sc, Ta, Ti, V, Y, and Zr can improve the plasticity. It is shown that the addition of Al, Bi, Nb, Sc, Ta, Ti, V, Y, and Zr elements to Cu_3_P by brazing companies can improve the processing efficiency of Cu_3_P, ensure the quality of its joints, as well as enhance the repairability and reliability of the product. And if companies want to increase the hardness, they can add the elements In, Bi, Ge, Hf, Pb, Sb, Si, and Sn to the Cu_3_P phase.

(3) The results of the density of states show that doping with In, Si, Sn, and Ge increases the covalent bonding against shear strain, and doping with Sc, Y, Ti, Zr, Nb, and Ta is more prone to form metallic bonding, which reduces the hardness of the system, which is in accordance with the results of the mechanical property calculations.

## Figures and Tables

**Table 1:** The formation energy of doped other elements.

	The Formation Energy of Doped (eV)	
	Substituted P	Substituted Cu
CuPAl	3.4878	1.1919
CuPIn	7.0751	5.0229
CuPSi	−1.2637	1.2642
CuPGe	0.9096	1.0031
CuPSn	3.4962	5.2153
CuPPb	1.0973	1.1961
CuPSb	1.0393	1.1533
CuPBi	1.138	1.248
CuPSc	1.0137	0.894
CuPY	1.1317	0.9915
CuPTi	0.9469	1.015
CuPZr	3.0571	−1.1363
CuPHf	0.9735	1.0212
CuPV	0.9821	0.8592
CuPNb	0.9468	0.7869
CuPTa	0.9229	0.777

**Table 2:** Comparison of lattice constant results for Cu_3_P.

	Calculated Values in This Paper			Literature Experimental Values [17]			Literature Calculated Values [18]		
Cu_3_P	a/Å	b/Å	c/Å	a/Å	b/Å	c/Å	a/Å	b/Å	c/Å
	6.974	6.974	7.199	6.959	6.959	7.143	6.96	6.96	7.18

**Table 3:** Comparison of lattice constant calculation results.

	a/Å	b/Å	c/Å	Volume/Å
CuPAl	7.0103	7.0103	7.316	311.3727
CuPIn	7.0936	7.0936	7.403	322.6141
CuPSi	6.968	6.9664	7.2143	304.4818
CuPGe	7.0344	7.0344	7.2201	309.4002
CuPSn	7.1849	7.0899	7.2743	319.2132
CuPPb	7.1557	7.1557	7.3446	325.6873
CuPSb	7.1252	7.1252	7.3133	321.5426
CuPBi	7.1748	7.1748	7.3642	328.3069
CuPSc	7.1025	7.1025	7.29	318.4796
CuPY	7.1861	7.1861	7.3758	329.862
CuPTi	7.0805	7.0805	7.2674	315.5243
CuPZr	7.0226	7.0226	7.5458	331.3888
CuPHf	7.1563	7.1563	7.3452	325.7642
CuPV	7.0196	7.0196	7.2049	307.4571
CuPNb	7.088	7.0880	7.2751	316.537
CuPTa	7.0887	7.0887	7.2758	316.6252

**Table 4:** Comparison of experimental and calculated ingredient content.

	P	Si	Sn	Cu
CuPSi	12.50 (11.67)	0.26 (2.12)	/	Allowance
CuPSn	11.38 (10.93)	/	6.42 (8.37)	Allowance

## Data Availability

The data presented in this study are available in the article.
